# Principal Characteristics of Affected and Unaffected Side Trunk Movement and Gait Event Parameters during Hemiplegic Stroke Gait with IMU Sensor

**DOI:** 10.3390/s20247338

**Published:** 2020-12-21

**Authors:** Jeong-Woo Seo, Seul-Gee Kim, Joong Il Kim, Boncho Ku, Kahye Kim, Sangkwan Lee, Jaeuk U. Kim

**Affiliations:** 1 Future medicine division, Korea Institute of Oriental Medicine, Daejeon 34504, Korea; jwseo02@kiom.re.kr (J.-W.S.); sgkim11@kiom.re.kr (S.-G.K.); jikim@kiom.re.kr (J.I.K.); secondmoon@kiom.re.kr (B.K.); kkh2@kiom.re.kr (K.K.); 2 Department of Internal Medicine and Neuroscience, College of Korean Medicine, Wonkwang University, Iksan 54538, Korea; sklee@wku.ac.kr; 3 Korean Convergence Medicine, University of Science and Technology, Daejeon 34054, Korea

**Keywords:** stroke, IMU sensor, hemiplegic gait, principal component analysis, gait event

## Abstract

This study describe the characteristics of hemiplegic stroke gait with principal component analysis (PCA) of trunk movement (TM) and gait event (GE) parameters by the inertial measurement unit (IMU) sensors: (1) Background: This process can determine dominant variables through multivariate examination to identify the affected, unaffected, and healthy lower-limb sides; (2) Methods: The study monitored forty patients with stroke and twenty-eight healthy individuals comprising the control group for comparison. The IMU sensors were attached to each subject while performing a 6 m walking test. Sixteen variables extracted from the measured data were divided into 7 GE and 9 TM variables explaining pelvis tilt, oblique, and rotation. (3) Results: The tilt range variables of the trunk movement on the affected and unaffected sides were lower than those of the healthy side; this showed between-group differences in various GE variables. For the healthy and affected sides, 80% of variances were explained with 2 or 3 PCs involving only a few dominant variables; and (4) Conclusions: The difference between each side leg should be considered during the development of a diagnosis method. This research can be utilized to develop functional assessment tools for personalized treatment and to design appropriate training protocols.

## 1. Introduction

A hemorrhage or thrombus affects arterial supply to the brain, leading to a stroke; this is then followed by neurological impairments such as damage in motor cells and pathways. Therefore, degradation or loss of motor functions occurs. Control problems in the central nervous system decrease muscle strength and generate spasms. The main reason for spasms is voluntary muscle contractions of abnormal magnitude in any muscle group. Consequently, there is no support for the limb segments during the stance phase, and foot swings during the swing phase are not performed normally [1].

Gait is a very important factor in functional ambulation in stroke patients and can be used as an indicator of the stroke level [2]. Functional gait assessment, dynamic gait index, and timed up and go tests are used for gait evaluation [3,4,5]. The results of these tests are indicators of the level of walking impairment when walking while performing a simple task. Various variables are used as parameters for gait evaluation, including time variables associated with conventional tasks, owing to the development of inertial sensors that can quantify dynamic functions [6]. The spatial and temporal variables can be calculated, such as the range of motion, walking speed, and event ratio. These variables can quantitatively evaluate diseases. These variables were used to study the functional evaluation of the patients with intervertebral disc disease through gait [7]; also, the walking characteristics of stroke patients are more precisely defined as quantitative measurements.

The description of kinematic changes in hemiplegic stroke patients varies. It is divided into the unaffected and affected sides. As asymmetry occurs, the stance phase becomes longer and the swing phase becomes shorter as the walking speed decreases. Changes occur in the hips with flexion and extension. At the initial contact, decreased hip flexion, increased hip flexion at toe-off, and decreased hip flexion during mid-swing occur. Knee flexion decreases and ankle plantar flexion increases during the initial contact and swing phase. The asymmetry of stroke patients is mainly discussed with respect to the movement of the trunk [8].

Trunk variability is a very important factor in walking. In particular, the left and right symmetries are important. In stroke patients, trunk symmetry is assessed by determining the symmetry ratio of the paretic and non-paretic sides and the asymmetry index or symmetry score, which is quantified relative to a calculated midpoint between the feet [8]. The symmetry ratio is an indicator of the difference between the unaffected and affected sides and can be used to confirm the effectiveness of rehabilitation therapy through observation of reduced asymmetry levels. Subsequently, individual status evaluations are required for treatment of both affected and unaffected sides. Furthermore, it is necessary to analyze the characteristics of each side to develop a new motor function evaluation method.

The difference can be confirmed by comparing the average values of the measured characteristic variables on each side. This can be confirmed by analyzing the variance of each sample. However, there is a limit to explaining the gait characteristics of each side. PCA can be used to identify the principal components (PCs) that have the most representative characteristics among a wide variety of parameters measured using inertial sensors. PCA is a method of linearly transforming data so that when the data are projected on one axis, the variance becomes the axis in order of increasing variance [9]. The data on each axis is where the most important components are located. This is an exploratory data analysis technique that analyzes multivariate data by understanding the structure of the covariance matrix.

A powerful property of PCA is that a majority of the variation may be explained by the first few PCs. Several previous studies identified the PCs among the gait parameters using PCA. One study measured kinematic and kinetic variables of spatial data when identifying the gait characteristics of stroke patients. The results confirmed the PCs related to velocity and those related to the symmetry between two joints of the lower limb [10]. Another study performed PCA for identifying the dominant variables from the gait-related parameters in spastic diplegic cerebral palsy children [11]. As such, the dominant variables can be determined from the data available through a multivariate examination performed to identify the affected, unaffected, and healthy lower-limb sides. In particular, it is necessary to determine the correlation between the measured muscle strength and the basis for the dynamic function in the case of the affected side. In other words, it is possible to identify meaningful representative variables from several variables that can be measured using one inertial measurement unit sensor, develop functional assessment tools for personalized care, and perform fundamental analysis at the basic stage using appropriate training protocols at the design level.

In this study, we compared the walking and trunk movements between the leg sides of hemiparesis stroke patients and the healthy control group. The purpose of this study was to examine the variables influencing the movements on each leg side through PCA, and to extract those variables with a strong explanatory power. In addition, the correlation between the PC variables and the muscle strength of the lower limbs on the affected side was examined; it was tried to confirm the evidence of the result.

## 2. Materials and Methods

The Wonkwang University Gwangju Hospital (WKUGH) outpatients or inpatients with hemiparesis due to stroke were selected. The inclusion criteria were a history of stroke within the previous 6 months and the ability to safely walk 5 m without physical assistance or unaided. Those who had difficulty walking due to other conditions such as musculoskeletal disease were excluded [12,13]. Subjects were in the Korean-National Institute of Health Stroke Scale (K-NIHSS) based minor stroke just before their return to daily life. Forty inpatients with stroke were recruited. Twenty-eight control group subjects, for comparison with patients, were selected as the subjects to perform normal gait in daily life. Study group characteristics are shown in Table 1. The K-NIHSS and manual muscle testing (MMT) of lower extremity scores were also measured. The method of this study was approved by the ethics committee, institutional review board (IRB) in WKUGH (WKIRB–18/8, 29 April 2018).

In order to perform quantitative measurements during the walking test, the G-walk system and BTS G-studio software (ver. 2.8.16.0, BTS Bioengineering Inc., Quincy, MA, USA) were used. The G-walk system is a gait analysis system that measures the center of mass using a wireless tri-axial accelerometer [12]. It was attached to the fifth lumbar vertebra of the subjects and tightened using Velcro elements. The subjects were asked to walk twice on the 6 m floor at a preferred daily walking speed. Sixteen variables were obtained from the measured data and the variables were divided as follows: gait event variables (cycle duration, step length, stance duration, swing duration, double support duration, single support duration, and strides elaborated), pelvis tilt symmetry (sagittal plane, minimum value, maximum value, and range), pelvis oblique symmetry (frontal plane, minimum value, maximum value, and range), and pelvis rotation symmetry (transversal plane, minimum value, maximum value, and range) [12].

Data calculations and statistical analyses were performed with MATLAB R2019b (MathWorks Inc., Natick, MA, USA). The two groups (affected/unaffected and healthy) were compared using independent *t*-tests. Within a patient’s legs, the affected and unaffected leg sides were compared using paired *t*-tests. Spearman’s correlations were used to investigate the relationship between lower-limb muscles in MMT and gait variables. PCA was used for data classification and data structure detection. This method was applied to extract the maximum variance from the data through a few orthogonal components called PCs [13]. A matrix was created from the sixteen variables obtained from the data for each group of the sampled population. The variables were normalized with the z-score. The criteria for variable selection with group descriptions are having a main component with a mean variance greater than 10 and a cumulative proportion of approximately 80% [14].

## 3. Results

There were significant differences in the stance, swing duration, and single support duration of gait-event-related variables. Comparison of results showed that the tilt range of the trunk movement on the affected and unaffected sides was significantly lower than that on the healthy side. There were no differences in obliquity and rotation during trunk movement. The gait cycle duration was low on the healthy side, double support duration on the affected side was lower than that on the healthy side, and single support duration on the unaffected and healthy sides was higher than that on the affected side. The stride elaborated on the healthy side was lower than that on the affected and unaffected sides (Table 2).

**Figure 1:**
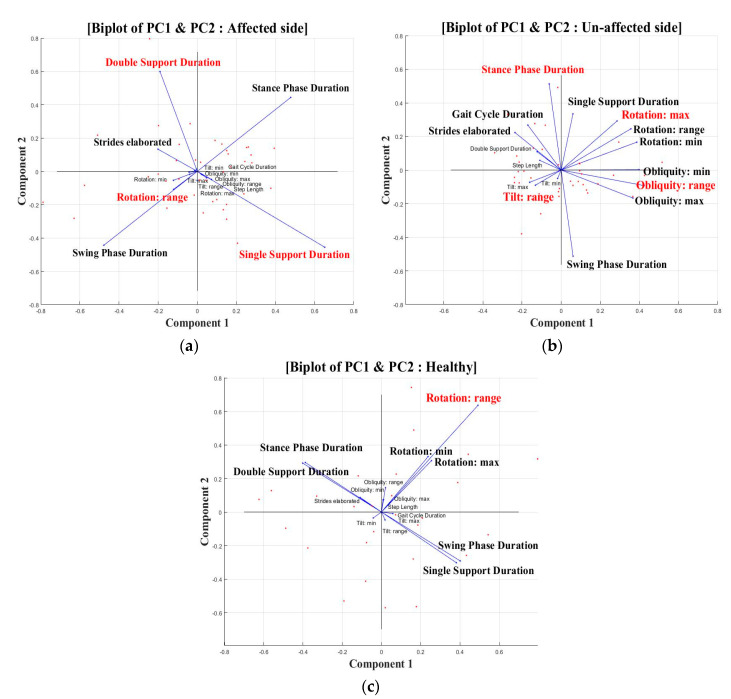
Biplots of selected features by PCs for (**a**) the affected side, (**b**) the unaffected side, and (**c**) healthy side.

The three, four, and two PCs were the last ones whose eigenvalues were larger than 10 and the cumulative percentage of the total inertia of the PC (PC scores) was approximately 80% in each leg side group. The PC variables selected using this criterion were the following (Table 3). The variables with high PC scores were plotted in the two simple PC1 and PC2 scatter plots (Figure 1).

The correlation results between the lower-limb muscles in the MMT and the gait event, and the trunk movement variables in the affected group shows that the maximum value of the obliquity is significantly correlated with the hip and knee muscle functions. The ankle muscle function was not correlated with the abovementioned variables (Table 4 and Table 5).

## 4. Discussion

The purpose of this study was to identify strongly explanatory variables for each leg side through PCA. In addition, the correlation between the descriptive variables and the muscle strength on the affected side was examined to check the effect of muscle strength on gait. There was no difference in the trunk movements, but there were differences in the gait cycle duration, stance and swing phase ratios, and double and single support durations between the affected and unaffected sides.

The gait cycle duration was approximately 0.15 s longer than that on the healthy side. In general, the stance and swing phases of a hemiplegic stroke patient have longer durations on both the affected and unaffected sides and account for a greater proportion of the gait cycle [1]. It is the same result as found in the previous study comparing the unaffected and the affected side gait parameters using an IMU sensor [15]. Moreover, the stance phase is both longer and occupies a greater proportion of the gait cycle on the unaffected side than on the affected side. We found that the duration of the stance phase on the unaffected side was approximately 2% longer. Furthermore, in the stance phase where weight shifting is performed, the weight is more dependent on the healthy side than on the affected side. A greater proportion of the gait cycle is spent for double support in hemiparetic walkers than in able-bodied individuals walking at normal speeds [16]. In this study, the double support duration on the affected side increased by 2%. Changes in the event rate on the affected side occur through activation of the unaffected side to compensate body support duration of affected side [17].

Additionally, stroke commonly results in trunk impairments associated with decreased trunk coordination and limited trunk muscle strength. These impairments in gait are caused by the pelvic step [8]. Three types of evaluations were performed to identify gait characteristics such as the pelvic step of hemiplegic stroke patients. The first one involved identifying the linear or angular variables corresponding to the kinematics of the trunk movement. In general, hemiplegic stroke patients have the following kinematic characteristics. In the frontal plane, the affected side shows enlarged lateral swaying of the lower and whole trunk during the stance phase [18]. In the sagittal plane, stroke patients tilt their pelvis on the affected side upward during the stance phase and downward during the swing phase [19]. Furthermore, in the transverse plane, the range of motion of the upper trunk is larger than that of the lower trunk. The second is trunk coordination. Representatively, the continuous relative phase is identified by examining the degree of dissociation of thoracic and pelvic segments in the transverse plane [20]. The third is trunk variability. Trunk symmetry refers to trunk displacements or accelerations between the affected and unaffected sides. Most previous studies showed increased trunk variability in stroke patients [21,22]. However, in this study, there was no statistical difference in the variability of all trunk movements except for the tilt range of motion (RoM). The tilt in the sagittal plane showed no difference in the minimum and maximum values, and the RoM of the stroke patients decreased by approximately 0.5°.

This result is in contrast with the excessive pelvic tilt occurring when compared with normal subjects. There are two main reasons for this. The first is the stroke severity of the study subjects. There is no difference in the maximum and minimum values of tilt because the patients had a mild stroke and could almost walk normally; the NIHSS for a minor stroke was only 0.9 ± 1.5. The purpose of this study was to identify the factors necessary for gait rehabilitation of patients at the stage just before returning to daily life. The results of this study indicated that there were no differences in the trunk movement between the unaffected and affected sides, but differences existed in the variables related to the walking event. The second reason is the rehabilitation stage. The patients in this study tilted their pelvis down on the affected side during the stance phase in the same direction as that of the healthy group. The direction of pelvic movement was the same as that of the healthy subjects and the RoM decreased. The influence of the compensatory trunk movement was reduced and the direction of movement was the same as that observed in a normal person; the influence could also be reduced if a mild stroke occurs at a level where no compensatory trunk movement occurs. A reduced RoM is expected owing to intrinsic trunk control deficits and reduced muscle strength. The rationale for this must be confirmed through additional follow-up studies. 

PCA was performed to identify the main components representing unaffected, affected, and healthy leg sides by combining gait event and trunk variables during walking. The results for the affected and healthy sides were reduced to several main components, but several variables classified as the main components based on the PC score were identified for the unaffected side. On the affected side, the single and double support durations of the gait-event-related variables and the rotation range in the trunk movements were found to be the explanation of the variation. The three-gait event and the trunk movement related variables could explain 90% of the variance of the affected side. The single and double support durations were significantly reduced on the affected side. The affected side lacks the strength to support the legs when walking [16]. Therefore, it is necessary to examine these major variables to confirm the recovery levels in rehabilitation. 

For the healthy leg side, explanation of the variation was confirmed in various variables. The obliquity range showed the highest PC score among the trunk movements, and the tilt range, rotation maximum, stance of the walking event, and swing phase duration were confirmed. In the healthy group, the rotation range variable was identified as the main component in both PC1 and PC2; the walking of a normal person could be explained by a rotation range of up to 90%. In addition, the obliquity, tilt, and rotation variables in all directions of the trunk were identified as the PCs of the unaffected side. This means that the gait pattern caused by intrinsic trunk control deficits was more apparent on the unaffected side than on the affected side. Therefore, the main clinical factor influencing the gait rehabilitation of stroke patients could be improved by treating both the affected and unaffected sides simultaneously [23]. Therapeutic methods may be utilized for improving neuroplasticity-based connectivity and muscle strength associated with intrinsic control deficits [1,24].

Among the kinematic variables on the affected side, it is necessary to determine the correlation between the muscle forces required for each lower-limb movement and select the appropriate strength improvement exercises in rehabilitation. For this reason, the correlation between the measured MMT and the affected side was examined in this study. As a result, trunk obliquity was significantly correlated (*p* < 0.05, r = 0.36) with hip and knee flexion and extension. This means that the oblique movement of the trunk can be estimated by measuring the muscle force involved in the hip and knee movements. However, ankle movements were not correlated; the muscle strength involved in ankle movement is less related to hip movements. This may be owing to the influence of the bi-articular muscles involved in the movement of two or more limbs. For example, the rectus femoris muscles are involved in flexing movements both at the hip and knee joints. Detailed electromyography measurements are required to determine why other lower-limb muscle strengths were not correlated with the various parameters identified in this study.

The main contribution of this study is to find out which of the variables of gait are the main variables that can explain the affected side, unaffected side and healthy legs. The limitation of this study is that the absolute level of the active ingredients is not presented; in addition, the severity of stroke patients of various levels has not been classified. This is a preliminary study to make an index for more quantitative and objective diagnosis of the affected side through further studies by synthesizing the results. We will use various classification analysis tools such as linear discriminant analysis, and support vector machines to differentiate between healthy control and stroke patients, and propose diagnostic criteria for patients with more diverse stroke levels through follow-up research.

## 5. Conclusions

To summarize, this study identified the dominant variables associated with the trunk movements and gait event parameters of the affected and unaffected sides during hemiplegic gait after a minor stroke. The variances of healthy and affected sides were explained by 2 or 3 PCs composed with only a few dominant variables; however, on the unaffected side, more diverse variables were identified as the PCs were based on their PC scores. The difference between each side leg should be considered during the development of a diagnosis method. This research can be utilized to develop functional assessment tools for personalized treatment and to design appropriate training protocols.

## Figures and Tables

**Table 1:** Characteristics of the study group.

Variable	Patients	Control
No.	40	28
Age (years)	65.1 ± 9.4	65.9 ± 7.8
Sex (women/men)	22/18	16/12
Hemiparetic side (left/right)	14/26	-
Time post-stroke (months)	3.0 ± 1.9	-
K-NIHSS	0.9 ± 1.5	-
MMT-lower extremity	38.8 ± 3.8/50	50/50
(affected/unaffected)		

**Table 2:** Gait variables of hemiplegic stroke patient’s affected/unaffected side and healthy control.

Gait Measure	Affected Side	Unaffected Side	Healthy (L&R Mean)
Gait event			
Gait cycle Duration (sec)	* 1.19 (0.15)	^%^ 1.19 (0.15)	*^,%^ 1.04 (0.08)
Step length (%)	49.99 (2.55)	49.89 (2.54)	50.00 (0.00)
Stance duration (%)	^#^ 60.19 (3.96)	^#^ 62.85 (5.37)	60.98 (2.19)
Swing duration (%)	^#^ 39.81 (3.98)	^#^ 37.16 (5.37)	39.02 (2.19)
Double support duration (%)	* 12.89 (3.60)	11.95 (2.51)	* 10.97 (2.14)
Single support duration (%)	*^,#^ 35.69 (5.02)	^#^ 38.33 (4.30)	* 39.04 (2.14)
Strides elaborated (no.)	* 11.93 (3.27)	^%^ 12.10 (3.07)	*^,%^ 9.45 (1.24)
Tilt			
Tilt: minimum (°)	1.49 (0.64)	1.49 (0.66)	1.86 (0.98)
Tilt: maximum (°)	0.72 (0.52)	0.74 (0.56)	0.89 (0.58)
Tilt: range (°)	* 2.20 (0.73)	^%^ 2.21 (0.79)	*^,%^ 2.74 (0.88)
Obliquity			
Obliquity: minimum (°)	1.02 (0.60)	1.08 (0.58)	1.15 (0.52)
Obliquity: maximum (°)	1.08 (0.57)	1.04 (0.58)	1.15 (0.51)
Obliquity: range (°)	2.09 (1.05)	2.12 (1.04)	2.30 (1.04)
Rotation			
Rotation: minimum (°)	3.17 (1.57)	3.27 (1.70)	3.60 (1.73)
Rotation: maximum (°)	3.33 (1.86)	3.30 (1.84)	3.55 (1.71)
Rotation: range (°)	6.50 (3.15)	6.56 (3.31)	(3.42)

^#^: Significant difference in values between stroke affected and unaffected side (paired *t*-test, *p* < 0.05, α = 0.05). *^, %^: Significant difference in values between stroke and healthy group (independent *t*-test, *p* < 0.05, α = 0.05).

**Table 3:** Principal component analysis of each group.

**Groups**	**PCs**	Highest PC Variable	PC Coefficient	PC Scores (%) (Variation Explained)
Affected side	PC1	Single support duration	0.65	41.7
	PC2	Double support duration	0.59	25.8
	PC3	Rotation: range	0.75	14.4
Unaffected side	PC1	Obliquity: range	0.43	28.2
	PC2	Stance/Swing phase duration	0.51/−0.51	18.4
	PC3	Tilt: range	0.63	13.1
	PC4	Rotation: maximum	0.37	10.9
Healthy side	PC1	Rotation: range	0.49	50.1
	PC2	Rotation: range	0.64	39.5

**Table 4:** Correlation between the lower-limb hip muscles in the MMT and the gait variables of hemiplegic stroke patient’s affected side.

Gait Measure	Hip					
	Flexion	Extension	Abduction	Adduction	Internal Rotation	External Rotation
Gait cycle duration	−0.30	−0.30	−0.30	−0.30	−0.30	−0.30
Step length	−0.00	−0.00	−0.00	−0.00	−0.00	−0.00
Stance phase duration	0.14	0.14	0.14	0.14	0.14	0.14
Swing phase	−0.14	−0.14	−0.14	−0.14	−0.14	−0.14
Double support duration	0.05	0.05	0.05	0.05	0.05	0.05
Single support duration	0.03	0.03	0.03	0.03	0.03	0.03
Stride elaborated	−0.24	−0.24	−0.24	−0.24	−0.24	−0.24
Tilt: min	0.05	0.05	0.05	0.05	0.05	0.05
Tilt: max	−0.14	−0.14	−0.14	−0.14	−0.14	−0.14
Tilt: range	−0.11	−0.11	−0.11	−0.11	−0.11	−0.11
Obliquity: min	0.06	0.06	0.06	0.06	0.06	0.06
Obliquity: max	* 0.36	* 0.36	* 0.36	* 0.36	* 0.36	* 0.36
Obliquity: range	0.23	0.23	0.23	0.23	0.23	0.23
Rotation: min	−0.11	−0.11	−0.11	−0.11	−0.11	−0.11
Rotation: max	−0.14	−0.14	−0.14	−0.14	−0.14	−0.14
Rotation: range	−0.15	−0.15	−0.15	−0.15	−0.15	−0.15

*: Significant in Spearman’s rho value (*p* < 0.05, α = 0.05), hip: gluteus medius/maximus.

**Table 5:** Correlation between the lower-limb knee and ankle muscles in the MMT and the gait variables of hemiplegic stroke patient’s affected side.

Gait Measure	Knee		Ankle	
	Flexion	Extension	Dorsi-Flexion	Plantar-Flexion
Gait cycle duration	−0.30	−0.30	−0.16	−0.16
Step length	−0.00	−0.00	−0.10	−0.10
Stance phase duration	0.14	0.14	0.27	0.27
Swing phase	−0.14	−0.14	−0.27	−0.27
Double support duration	0.05	0.05	−0.16	−0.16
Single support duration	0.03	0.03	0.34	0.34
Stride elaborated	−0.24	−0.24	−0.23	−0.23
Tilt: min	0.05	0.05	0.15	0.15
Tilt: max	−0.14	−0.14	−0.33	−0.33
Tilt: range	−0.11	−0.11	−0.15	−0.15
Obliquity: min	0.06	0.06	0.05	0.05
Obliquity: max	* 0.36	* 0.36	0.30	0.30
Obliquity: range	0.23	0.23	0.19	0.19
Rotation: min	−0.11	−0.11	−0.18	−0.18
Rotation: max	−0.14	−0.14	−0.10	−0.10
Rotation: range	−0.15	−0.15	−0.15	−0.15

*: Significant in Spearman’s rho value (*p* < 0.05, α = 0.05), knee: quadriceps femoris, ankle: ankle flexor.
